# Comparison of Influenza and SIV Specific CD8 T Cell Responses in Macaques

**DOI:** 10.1371/journal.pone.0032431

**Published:** 2012-03-05

**Authors:** Sinthujan Jegaskanda, Jeanette C. Reece, Robert De Rose, John Stambas, Lucy Sullivan, Andrew G. Brooks, Stephen J. Kent, Amy Sexton

**Affiliations:** 1 Department of Microbiology and Immunology, University of Melbourne, Melbourne, Victoria, Australia; 2 School of Medicine, Deakin University, Waurn Ponds, Victoria, Australia; University of Pittsburgh, United States of America

## Abstract

Macaques are a potentially useful non-human primate model to compare memory T-cell immunity to acute virus pathogens such as influenza virus and effector T-cell responses to chronic viral pathogens such as SIV. However, immunological reagents to study influenza CD8^+^ T-cell responses in the macaque model are limited. We recently developed an influenza-SIV vaccination model of pigtail macaques (*Macaca nemestrina*) and used this to study both influenza-specific and SIV-specific CD8^+^ T-cells in 39 pigtail macaques expressing the common Mane-A*10^+^ (Mane-A01*084) MHC-I allele. To perform comparative studies between influenza and SIV responses a common influenza nucleoprotein-specific CD8^+^ T-cell response was mapped to a minimal epitope (termed RA9), MHC-restricted to Mane-A*10 and an MHC tetramer developed to study this response. Influenza-specific memory CD8^+^ T-cell response maintained a highly functional profile in terms of multitude of effector molecule expression (CD107a, IFN-γ, TNF-α, MIP-1β and IL-2) and showed high avidity even in the setting of SIV infection. In contrast, within weeks following active SIV infection, SIV-specific CD8^+^ effector T-cells expressed fewer cytokines/degranulation markers and had a lower avidity compared to influenza specific CD8^+^ T-cells. Further, the influenza specific memory CD8 T-cell response retained stable expression of the exhaustion marker programmed death-marker-1 (PD-1) and co-stimulatory molecule CD28 following infection with SIV. This contrasted with the effector SIV-specific CD8^+^ T-cells following SIV infection which expressed significantly higher amounts of PD-1 and lower amounts of CD28. Our results suggest that strategies to maintain a more functional CD8^+^ T-cell response, profile may assist in controlling HIV disease.

## Introduction

Chronic viral pathogens such as HIV pose a major challenge to immune control. CD8 T cell responses partially control viral replication in both the acute and chronic phase of HIV and SIV infections. Evidence demonstrating the partial role of CD8 T cells in HIV/SIV include: depletion of CD8 T-cells in SIV-infected macaques increasing viral replication [1], [2], [3], [4], control of viral replication coinciding with the expansion of HIV/SIV-specific CD8 T cells [5], [6], immune pressure exerted by CD8 T cells leads to viral escape [7] and MHC alleles such as HLA-B*57 and HLA-B*27 being overrepresented long term non-progressor subjects [8], [9], [10], [11]. Although CD8 T cell responses are clearly important, the key characteristics of a protective CD8 T-cell response remain rather poorly defined. Numerous HIV vaccine studies show that the magnitude of this response correlates weakly with protection [2], [12], [13]. Recent studies have therefore included the measurements of quality and avidity. Quality of the response is commonly measured by breadth of expression of effector molecules such as IFN-γ, TNF-α, CD107a, IL-2 and MIP-1β [14], [15], [16] and avidity as exhibited by ability of MHC class I tetramer to dissociate over time [17], [18]. High avidity HIV-specific CD8 T cells have recently been shown to be more effective at clearing virus infection [13], [19]. In addition, other characteristics such as the memory phenotype, and kinetics of the CD8 T cell response are also likely to be very important [20], [21]. HIV-specific CD8 T cells present during chronic infection tend to express an “exhausted” phenotype with high PD-1 and low CD28 expression and are unable to proliferate in response to high concentrations of antigen [22], [23], [24], [25], [26].

A key problem with studying HIV-specific CD8 T cells is that these responses are generally unable to prevent the establishment of chronic infection. This contrasts with CD8 T cell responses to acute viral infections, such as influenza, where the CTL response is clearly linked to assisting the resolution of infection [27]. Lessons on immune control can likely be learnt from studies of acute infections where the CD8 T cell response assists in resolving the infection. There are however only a limited number of studies that have compared memory T-cell response to resolve acute viral infection to effector T-cell response to a chronic viral pathogen such as HIV. One such study that has investigated the characteristics of T-cells in response to HIV and influenza in humans is Betts *et. al.* using intracellular cytokine staining techniques of CD8 T cell responses following stimulation with peptide pools [14]. In this study it was shown that effector molecule production by HIV-specific CD8 T cells is an important factor in limiting HIV viral load. Furthermore, the total memory influenza-specific CD8 T cells from subjects with progressive HIV infection can express multiple effector molecules whereas HIV-specific CD8 T-cells in the same individuals are poorly functional [14]. Elucidating the differences between the CTL responses to these different viruses should provide insights into what qualities generate an effective CD8 T cell responses. However such comparative studies of influenza and HIV responses in humans is difficult as the timing of either influenza or HIV infection cannot be defined or controlled. Macaques are a well-established model for HIV infection and are increasingly used as a model for influenza infection [28], [29], [30], [31], [32]. However reagents for accurately studying CD8 T cell responses to influenza infection in macaques, such as MHC class I tetramers, are currently non-existent.

Advances in the SIV-pigtail macaque (*M. Nemestrina*) model of HIV infection have led to the characterization of the MHC class I alleles and the mapping of SIV CD8 T cell epitopes. This has in turn led to the generation of MHC I tetramer reagents to study SIV-specific CD8 T cells [33], [34], [35]. Although MHC class I tetramers have been developed for both humans and the mouse model of influenza infection, this is not the case for the non-human primate or ferret models of influenza infection where there is a lack of mapped influenza-specific responses [22], [36]. Identifying influenza CD8 T cell epitopes restricted by common MHC I alleles in this model will enable the production of influenza-specific tetramers. Since macaques are the only model in which both influenza and SIV can be studied simultaneously, developing influenza tetramer reagents will facilitate characterizing of the quality of the memory influenza-specific CD8 T cell responses in pigtail macaques and enable more precise comparisons to be made between the quality of influenza- and SIV-specific CD8 T cells. Such advances would in turn allow an improved analysis of functional characteristics that make up a robust antiviral immune response in order to apply this to future HIV vaccine strategies. We therefore developed important immunologic reagents and then studied the quality of the memory influenza-specific CTL responses after influenza infection in comparison to SIV-specific CTL responses either after vaccination or during chronic SIV infection.

## Materials and Methods

### Animals

We studied a total 39 pigtail macaques (*Macaca nemestrinca*) for CD8 T-cell immune responses to SIV and influenza viruses. All animals expressed the MHC class I allele Mane-A*10 (recently renamed Mane-A1*08401) as confirmed using allele specific PCR reactions and/or high-throughput sequencing [37], [38], [39]. The 39 pigtail macaques studied comprised (Table 1): 17 macaques inoculated with control influenza A viruses (n = 5) or influenza A viruses expressing SIV-CTL epitopes (n = 12, see below for description of viruses, 2 animals previously reported [28]); 8 previously reported SIV-infected Mane-A*10+animals infected with influenza viruses after SIV infection [28]; 8 influenza-uninfected Mane-A*10+control animals; and an additional 6 Mane-A*10+animals previously reported that were administered DNA and Fowlpox virus expressing SIV-Gag antigens to analyze SIV-specific CTL responses in the absence of SIV infection [40]. Prior to any procedures animals were anesthetized intramuscularly with ketamine (10 mg/kg). All studies were approved by the relevant animal ethics committees.

**Table 1:** Mane-A*10+ pigtail macaques studied for Influenza- and SIV-specific CD8 T cell responses.

Group	n	Influenza Infection	Influenza recombinants used	SIV infection	Comment	Reference
Influenza-SIV vaccine trial	17	PR8 twice, X31 twice over 17 weeks (see Fig. 4).	Wild type influenza (n = 5), influenza expressing* 3 SIV CTL epitopes* (n = 10), influenza expressing one SIV CTL epitope (n = 2)	SIV_mac251_ 25 weeks after first Influenza inoculation	2 vaccinees previously reported in Sexton et al [28]	
Influenza inoculation of SIV+ macaques	8	PR8 twice, X31 twice over 18 weeks, 1–2 years after SIV infection	influenza expressing one SIV CTL epitope (n = 8)	SIV_mac251_ 1–2 years prior to influenza inoculation		[28]
Naïve macaques	8	None	NA	None	Used to study specificity of influenza specific MHC I tetramer in absence of influenza infection	
SIV vaccinated macaques	6	None	NA	None	Macaques received DNA and Fowlpoxvirus prime boost vaccines**. Used to study SIV CTL responses in the absence of SIV infection	[39], [44]

* All SIV CTL epitopes were minimal epitopes expressed from within the stalk of influenza neuraminidase protein.

** The DNA and fowlpoxvirus vaccines expressed full length SIV Gag and Pol proteins from standard promoters.

### Recombinant influenza-SIV constructs

The recombinant influenza A viruses used were generated using an eight-plasmid reverse genetics system as previously outlined [28], [41], [42], [43], [44]. Briefly, the DNA construct contained eight influenza virus segments including a genetically manipulated NA segment containing one Mane-A*10 restricted CD8 T cell epitope sequence; KP9 (SIV Gag_164–172_), KVA10 (SIV Tat_114–123_) or KSA10 (SIV Tat_87–96_). The CD8 T cell epitopes were inserted separately into the NA stalks using the recombinant PCR techniques [42]. Two mouse-adapted strains of influenza A virus were used in the study X-31 (H3N2, A/HKx31) and PR8 (H1N1, A/Puerto Rico/8/1939), these viruses share the same internal gene segments however differ in their surface glycoprotein HA and NA genes. 3 separate constructs of each virus (either PR8 and X-31) were administered to the vaccine animals. The retention of the correctly inserted peptide epitope within the expanded virions was confirmed by sequencing prior to vaccination.

### Vaccination and challenge of pigtail macaques

Recombinant and wild-type influenza viruses were administered to animals by combined intratracheal and intranasal administration of 10^8^ PFU of influenza virus in PBS as previously described [28]. Successful infection with influenza viruses was confirmed in all animals by serology and influenza virus RNA recovery in serials swabs of the upper respiratory tract during infection, kindly performed by Drs K Laurie and A Hurt at the WHO Collaborative centre for influenza in Melbourne. Animals were infected with a 10^4^ TCID_50_ dose of SIV_MAC251_ either intrarectally or intravaginally as previously described [45] (kindly provided by Dr N Miller at the NIH). SIV infection was confirmed in all animals by monitoring SIV plasma RNA levels as previously described [28].

### Intracellular cytokine staining (ICS) assays

The ICS assays involved the addition of 1 µg/ml of co-stimulatory molecules anti-CD28 and anti-CD49d (Becton Dickinson [BD], San Jose, CA), 5–10 µg/ml of Brefeldin A (Sigma, St. Louis, MO, USA) and/or 5 µg/ml Monesein (eBioscience, San Diego, CA) to 235 µl of whole blood. Specific peptide antigens were also added at a concentration of 1 µg/ml unless otherwise stated. Appropriate controls were set up with an equivalent concentration of dimethyl sulfoxide (DMSO) (Sigma) and 1 µg/ml of Staphylococcal enterotoxin B (SEB, Sigma). Where necessary anti-CD107a FITC or APC-Cy7 (H4B4) (BD, San Jose, CA) was also added prior to stimulation. Cells were incubated at 37°C with 5% CO_2_ for 5.5 hours unless otherwise indicated. For assays gating on tetramer+ cells, Mane-A*10 tetramers were added for 40 minutes at room temperature in the dark. Following this, cells were incubated with surface antibodies CD8-APC-H7 (SK1), CD4-PeCy7 (L200), and CD3-Pacific Blue (SP34-4, all BD) for 30 minutes at room temperature in the dark. Red blood cells were then lysed using 1× BD FACs™ Lysing Solution for 10 minutes and white blood cells permeabilised using 1× FACs™ Permeabilising Solution 2 for 10 minutes (BD). Finally, cells were incubated at room temperature for 1 hour with antibodies for the intracellular cytokines IFNg-AF700 (B27), TNF-α-PE-Cy7 (mAb11), IL-2-PerCP-Cy5.5 (MQ1-17H12), and MIP-1β-PE (D21-1351, all BD). Cells were then washed and fixed with 1% paraformaldehyde (Sigma). Acquisition was performed on the LSRII flow cytometer (BD) with 1×10^6^ lymphocyte events generally collected. Samples were analysed using FlowJo Version 9.1 (TreeStar, Ashland, USA). Polyfunctional analysis was performed using PESTLE and SPICE software (kindly supplied by Dr M Roederer, Vaccine Research Centre, NIH).

### Generation of CD8 T cell lines

Influenza-specific CD8 T cell lines were generated as described previously [35]. Briefly, fresh PBMCs were separated into either *stimulators* (1×10^6^) or *responders* (2×10^6^, a 1∶2 ratio). Responder cells were resuspended in RPMI plus 15% Fetal calf serum (RF-15) and placed at 37°C with 5% CO_2_ in a 24-well plate while stimulators were prepared. Stimulator cells were resuspended in 1 mL RF-15 supplemented with 1 µg/ml RA9 peptide and incubated at 37°C with 5% CO_2_ for 90 minutes. Cells were then washed 3 times with RF-15 media and resuspended to a volume of 100 µl that was then added to the responder cells. At day 3 and day 5 cells were given fresh RF-15 media containing 50 U/ml recombinant human interleukin-2 (IL-2) and 10 ng/ml human interleukin-7 (IL-7). All cultures were restimulated at day 7 with peptide pulsed irradiated autologous PBMCs and checked for specificity via ICS assay at day 10 and 20.

### Stimulation assay to establish MHC restriction

Stable cell lines of C1R cells expressing either Mane-A*10 or Mane-B*02 were generated as previously described [35]. RA9-specific T cells (responders) cultured for 3 weeks were washed and resuspended in RF-15 media. Cells were counted and separated in a 24-well plate to contain 2×10^6^ cells per well. The C1R cells (antigen presenting cells) with either Mane-A*10, Mane-B*02 or no MHC class I allele, were pulsed with 1 µg/ml RA9 peptide or DMSO (negative control) and incubated at 37°C with 5% CO_2_ in a 24-well plate for 30 minutes. The cells were then washed twice and 1×10^6^ stimulator cells were added to each of the individual wells. At this point, 10 µg/ml Brefeldin A (Sigma), anti-CD28 and anti-CD49d costimulatory molecules (BD) were added to each of the wells and incubated at 37°C with 5% CO_2_ for 6 hours. Samples were then processed as in the ICS assay.

### Tetramer development

The Mane-A*10-RA9 tetramer reagent was produced using the JA5 expression plasmids encoding the Mane-A*10 and monkey β_2_M as previously described [46]. The vector encoding Mane-A*10 also contained an in-frame fusion of the substrate sequence for biotinylation by the enzyme BirA. The expression, purification and refolding of the Mane-A*10 and monkey β_2_M with RA9 peptide was performed as previously described [46]. Briefly, proteins were expressed in *E. coli* BL21 cells and were solubilised from inclusion bodies. The proteins were refolded with synthetic RA9 peptide (Genscript, New York, USA) and resulting Mane-A*10-RA9- β_2_M complexes were purified by anionic exchange and gel filtration chromatography. Purified complexes were biotinylated and tetramerised with the addition of streptavidin-APC (Invitrogen, CA, USA).

### PD-1 and memory marker staining

Thawed PBMCs were stained Live/Dead Fixable Aqua Dead Cell Stain (Invitrogen, CA, USA) for 30 mins at room temperature in the dark. Subsequently, the sample was stained with anti-CD3 Pacific Blue (SP34-4), Anti-CD8 APC-H7 (SK1), Anti-CD4 PE-Cy7 (L200), Anti-CD28 PerCP-Cy5.5 (L293), Anti-CD95 FITC (DX2, all BD) and anti-CD279 PE (PD-1, J105, eBioscience). Samples were then stained with a titration-optimized volume of KP9-APC, KVA10-APC or RA9-APC tetramer for 40 mins at room temperature in the dark. Then washed with 1×PBS and fixed in 1% formaldehyde and acquired on LSRII flow cytometer (BD) with 1×10^6^ lymphocyte events generally collected.

### Tetramer association assay

Thawed PBMCs were stained with Live/Dead Fixable Aqua Dead Cell Stain (Invitrogen) for 30 mins at room temperature in the dark. Subsequently, the sample was stained with anti-CD3 Pacific Blue (SP34-4), anti-CD8 APC-H7 (SK1), Anti-CD4 PE-Cy7 (L200), Anti-CD28 PerCP-Cy5.5 (L293) and Anti-CD95 FITC (DX2, all BD). Samples were then separately incubated with excess concentrations of KP9-PE and RA9-APC tetramer for either 1, 3, 10 or 30 mins at room temperature in the dark. Following incubation all samples were washed with 1× PBS and fixed with 1% formaldehyde. Samples were acquired on LSRII flow cytometer (BD) with 1×10^6^ lymphocyte events generally collected.

## Results

### Influenza NP-specific CD8 T cell response

To analyze influenza-specific CD8 T cells, we first studied 17 Mane-A*10+ pigtail macaques that were infected intranasally and intracheally with unmodified influenza viruses or influenza viruses expressing Mane-A*10-restricted SIV CTL epitopes. Influenza inoculation used alternating doses of attenuated mouse-adapted H1N1 (PR8) and H3N2 (X31) strains to ensure efficient antigen presentation. The infections were asymptomatic but animals seroconverted and influenza RNA was recovered from serial swabs of the upper respiratory tract 2 days after inoculation.

**Figure 1:**
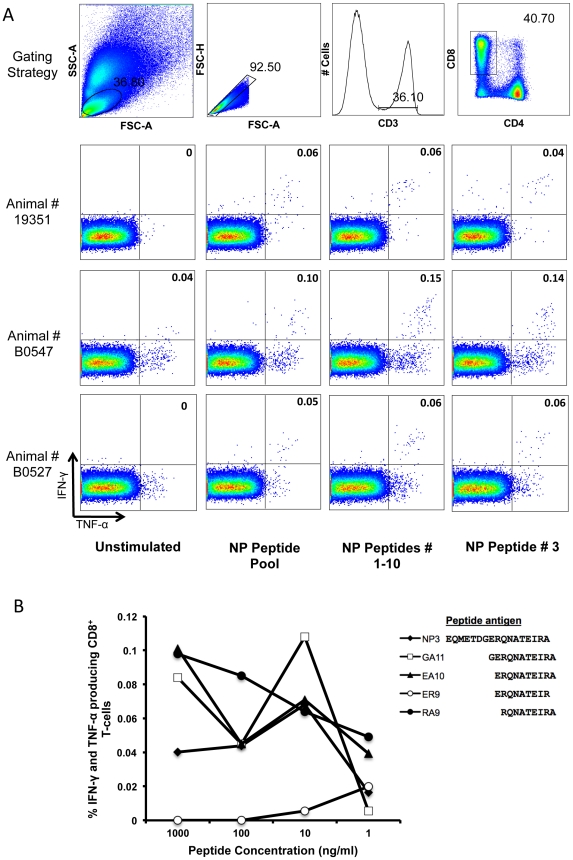
Mapping of influenza-specific CD8 T cell responses. (**A**) Gating strategy and IFN-γ and TNF-α expression of CD8 T cells in response to influenza nucleoprotein (NP) from influenza vaccinated 19351, B0547 and B0527, were investigated first by stimulating whole blood with a pool of 98 17-mer peptides overlapping by 12 amino acids (NP peptide pool), then to pools of 10 17-mers and finally to individual 17-mers. A common response within the first 10 17-mers (NP peptides 1–10) was mapped to the third nucleoprotein peptide (NP peptide #3). (**B**) Peptide titration performed on the influenza-vaccinated animal B0547 at day 77 following the initial influenza vaccination. Whole blood was stimulated with 29 peptides (7 11-mers, 9 10-mers, 11 9-mers and 2 8-mers) at diminishing dilutions and responses measured by ICS assay. Peptides yielding responses above background are shown together with a representative negative peptide (ER9).

To identify common influenza-specific CD8 T cell epitopes, an intracellular cytokine-staining (ICS) assay was first used to detect IFN-γ and TNF-α expression from CD8 T cells responding to influenza nucleoprotein (NP) overlapping peptides (Figure 1). The 3 animals (19351, B0547 and B0527) that had the highest responses to the whole NP peptide pool at 14 days post initial vaccination with influenza were selected to map influenza NP-specific CD8 T cell epitopes. The 98 overlapping 17-mer peptides within the NP peptide pool were divided into 10 sequential sets of 10 17-mers peptides and used to stimulate whole blood from the selected influenza vaccinated animals in an ICS assay. The response in all three animals was primarily detected in the 1–10 peptide set (spanning NP amino acids 1 to 62, NP_1–62_, Figure 1a). The three animals were then screened for CD8 T cell responses to each individual 17-mer peptide within the NP_1–62_ peptide set and each animal was found to respond to a single 17mer peptide NP_10–27_, termed NP3 (Figure 1a).

### Fine mapping of NP-specific CD8 T cell response

Fine mapping was subsequently undertaken to determine the minimal CD8 epitope within the NP_10–27_ 17mer NP3 peptide. Overlapping 11-mer, 10-mer and 9-mer peptides spanning NP_10–27_ were titrated in an ICS assay with one animal (B0547). Cytokine responses were demonstrated after stimulation with peptides GA11 (NP_16–27_), EA10 (_NP15–27_) and RA9 (_NP18–27_) (Figure 1b). As the amino acid sequences of these peptides overlap and the most prominent response was elicited by peptide RA9 we concluded that the RA9 peptide most likely contained the minimal CD8 epitope. This was further confirmed by subsequent mapping assays with peptides spanning the C-terminal border of RA9 as well as 8-mer peptides within RA9, all of which elicited substantially lower responses than did RA9. Concordant results for fine mapping were also obtained in another influenza-vaccinated animal (B0527, data not shown). Once RA9 was determined as a minimal CD8 T cell epitope, this specific response was analyzed in other influenza-vaccinated animals. Thirteen of the seventeen influenza vaccinated animals demonstrated a RA9-specific CD8 T cell response measuring IFN-γ+/TNF-α+ by ICS assay, ranging from 0.02–0.4%.

### Polyfunctional analysis of influenza and SIV CD8 T cell responses

**Figure 2:**
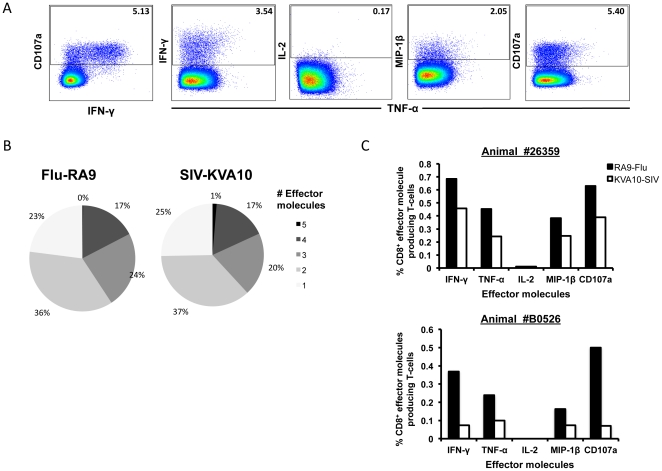
Comparison of polyfunctional influenza and SIV-specific CD8 T cell responses following recombinant influenza-SIV vaccination. Polyfunctional ICS assay was performed on whole blood from the animal 26359 at day 133 after initial influenza vaccination stimulating with either RA9 or KVA10 peptides. (**A**) Gating strategy on SEB stimulated sample (**B**) Summary of functional profile; the proportions of the number of effector molecules produced by cells when stimulated with either RA9 or KVA10 peptide. (**C**) The frequency of total effector molecules produced by RA9 (black bars) and KVA10 (white bars) specific CD8 T cells for animals 26359 and B0526.

In animals inoculated with recombinant influenza viruses expressing SIV CTL epitopes, comparisons could be made between the influenza-specific and SIV-specific CD8 T cell responses following vaccination. We used a polyfunctional ICS assay detecting the effector molecules IFN-γ, TNF-α, MIP-1β, IL-2 and CD107a, to compare the quality of influenza- and SIV-specific CD8 T cells. Combinatorial analysis was performed on cells stimulated with the newly mapped influenza NP CD8 T cell epitope RA9 or the SIV CD8 T cell epitope KVA10 peptides. Responding CD8 T cells were separated into distinct subsets based on the combination of effector molecules they produced (Figure 2a).

The number of effector molecules expressed by the responding CD8 T cells (their “polyfunctionality”) was similar for both the response to influenza RA9 and SIV KVA10 after the recombinant influenza-SIV infection, with the majority of responding CD8 T cells producing two effector molecules (Figure 2b). We also analyzed 2 other macaques with robust Flu or SIV-specific CTL response after Influenza-SIV vacation that permitted reliable division of the total response into multiple segments. We found the distribution of effector molecules expression was similar to the example shown in Fig. 2b. Overall, the mean (±SD) of 5, 4, 3, 2, 1 effector molecule expression was 1.0% (±0.8%), 18.5% (±5.2%), 21.8% (±2.6%), 38.8% (±3.6%) and 20.0% (±7.4%) respectively in all animals assessed. The pattern of individual effector molecule production by responding cells showed that the number of influenza RA9-specific CD8 T cells producing effector molecules were similar to that of SIV KVA10-specific CD8 T cells producing effector molecules across 2 animals with both influenza and SIV-specific CTL responses (Figure 2c). This is consistent with the 2 epitopes being expressed from the same construct. The majority of responding cells produced the anti-viral cytokine IFN-γ or the degranulation marker CD107a. Interestingly, minimal or no IL-2 was detected in response to either antigen (detected for the SEB positive control, data not shown) and therefore no CD8 T cells produced all five effector molecules. As such we did not study IL-2 production in subsequent experiments.

### MHC I restriction of influenza-RA9 CTL response

**Figure 3:**
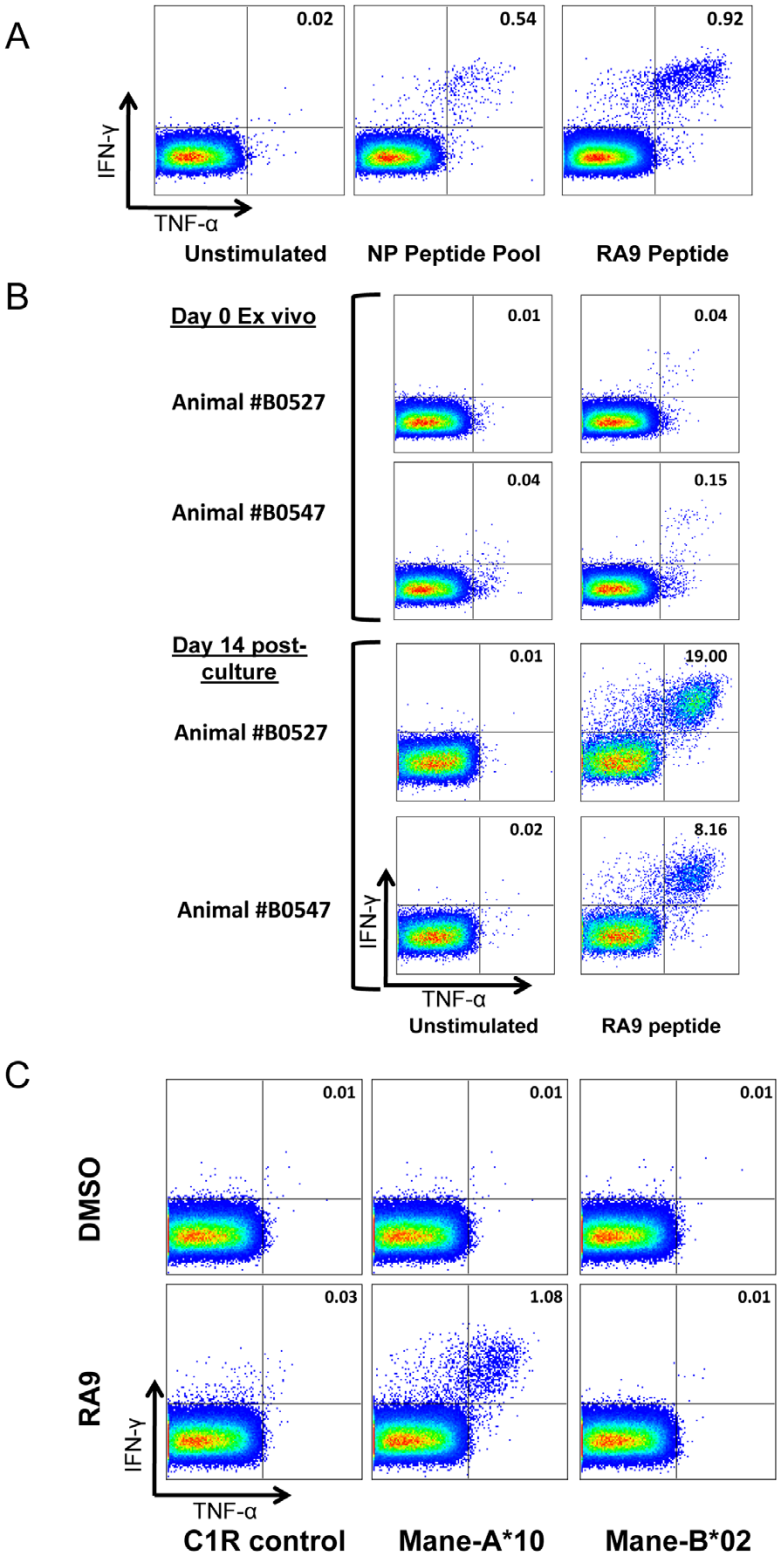
Restriction of RA9 CD8 T cell epitope to Mane-A*10. (**A**) CD8 T cell response to influenza RA9 peptide in influenza vaccinated animal 26359 (**B**) Expansion of RA9 specific CD8 T cells. RA9-specific CD8 T cell response in fresh blood is shown in comparison to a 2 week *in vitro* expansion as described in methods. (**C**) Mane-A*10 restriction of RA9 response. Transfected (Mane-A*10 or Mane-B*02) and untransfected C1R cells were pulsed with either DMSO or RA9 peptide. These C1R cells were incubated separately with *in vitro* cultured RA9-specific CD8 T cells and IFN-γ and TNF-α expression measured.

The influenza-inoculated animals studied all shared the MHC-I allele Mane-A*10 and we therefore investigated whether the influenza RA9 CTL response was restricted by this allele. The relatively low levels of memory RA9-specific CD8 T cells in blood necessitated expansion of these antigen-specific CD8 T cells for the definitive MHC restriction experiments (Figure 3a). We cultured PBMC *in vitro* in the presence of RA9 peptide-pulsed autologous PMBCs, IL-7 and IL-2 from three influenza vaccinated animals (B0527, 19351 and B0547) at day 91 post initial vaccination. In fresh blood, the frequency of RA9-specific CD8 T cells producing IFN-γ and TNF-α was approximately 0.04% for animal B0527 (Figure 3b) and 0.15% for animal B0547. Following the two-week culture, RA9-specific CD8 T cells expanded to frequencies of 19% for B0527 and 8.16% for B0547 (Figure 3b). This not only confirmed the presence of RA9-specific CD8 T cells in the blood but also facilitated in the restriction of this response to a given MHC class I allele.

To formally MHC restrict the RA9-specific CD8 T cells, untransfected control C1R cells and C1R cells stably transfected with full-length *M. nemestrina* MHC class I cDNA of either Mane-A*10 or the irrelevant Mane-B*02 [35] were pulsed with RA9 peptide or DMSO (negative control). These antigen-presenting cells were incubated with cultured RA9-specific CD8 T cells from animal B0527. An RA9-specific CD8 T cell response of 1.08% of CD8 T cells expressing both IFN-γ and TNF-α was detected from the RA9 pulsed Mane-A*10-transfected C1R cells. The control C1R cells and the irrelevant Mane-B*02-transfected C1R cells pulsed with either DMSO or RA9 peptide showed a RA9-specific CD8 T cell response of less than a 0.03% (Figure 3c). The results demonstrate that Mane-A*10 restricts the influenza RA9 CTL response.

### Generation and validation of Mane A*10-RA9 tetramer

**Figure 4:**
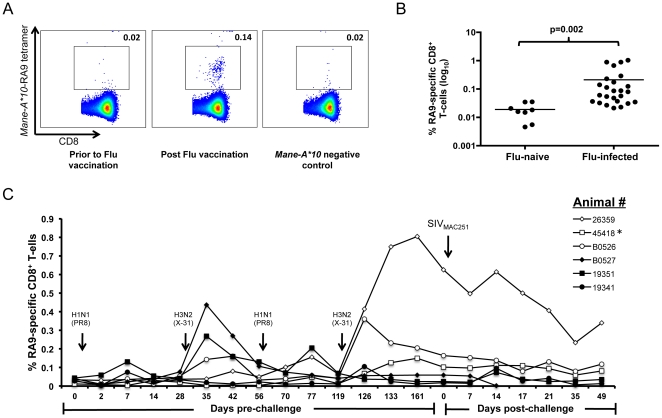
CD8 T cell response in influenza-vaccinated animals by Mane-A*10-RA9 tetramer. (**A**) Mane-A*10-RA9 tetramer was used at a 1∶400 dilution on samples from either animal 45418 at influenza vaccinated time-point (day 133) or influenza naïve time-point (day 0), or from an influenza unvaccinated Mane-A*10 negative animal. (**B**) The frequency of CD8^+^ Mane-A*10-RA9 tetramer positive cells were compared in all influenza vaccinated animals (n = 25) to 13 influenza naïve macaques all samples were 14 days post-final vaccination with influenza virus. All samples were background corrected and the p-value for the difference between the two groups determined using an unpaired T-test. (**C**) Thawed PBMC samples (day 0–126) or whole blood (day 126 onwards) from animals with generally robust influenza-RA9 tetramer responses on initial testing (26359, B0526, B0527, 45418, 19351 and 19341) were measured for frequency of RA9-specific CD8 T cells via tetramer staining (results are background corrected). Animals were vaccinated with influenza (either X-31 or PR8 as indicated) at day 0, 28, 56 and 119, then intravaginally challenged at day 175 (Day 0 post-challenge) with SIV_mac251_. All animals became SIV infected with the exception of 45418(*).

Confirmation of the MHC restriction of influenza RA9-specific CD8 T cells by Mane-A*10 led us to generate an MHC-I tetramer to enable further study of the influenza-specific response. A bacterial expression system was used to produce Mane-A*10 heavy chain and pigtail macaque β2M protein which were refolded with the RA9 peptide to form a stable MHC-peptide monomer [46]. The monomer was biotinylated and combined with a streptavidin-APC tag and formed a stable MHC class I tetramer. We evaluated the ability of the Mane-A*10-RA9 tetramer to bind and recognize RA9-specific CD8 T cells. Mane-A*10-RA9 tetramer staining of frozen PBMCs demonstrated a distinct RA9-specific CD8 T cell population (0.14% of CD8 T cells) post vaccination (Figure 4a). No distinct Mane-A*10-RA9 tetramer positive population was observed in the same animal prior to influenza vaccination or in an influenza naive Mane-A*10 negative animal (Figure 4a). To analyze the specificity of this reagent we studied Mane-A*10-RA9 tetramer staining of peripheral blood cells from 25 Mane-A*10+ influenza inoculated pigtail macaques and 8 influenza-uninfected animals. The influenza infected animals had significantly higher frequencies of Mane-A*10-RA9 tetramer positive cells than uninfected animals (Figure 4b).

The generation of the Mane-A*10-RA9 tetramer allowed us to analyze influenza-specific CD8 T cells in macaques longitudinally. We studied frequencies of RA9-specific CD8 T cells following multiple influenza inoculations. An increase in RA9-specific CD8 T cells was observed for most animals following the first infection with H1N1 (PR8) influenza which was boosted following inoculation with H3N2 (X-31) influenza at day 28; with the number of RA9-specific CD8 T cells in animal B0527 increasing from 0.08% at day 28 to 0.44% at day 35 (Figure 4c). The frequency of RA9-specific CD8 T cells further increased after the fourth influenza inoculation at day 119. Following infection with SIV_mac251_ at day 175, a slow decline in the frequency of RA9-specific CD8 T cells was generally observed in the influenza vaccinated animals. This was also seen for animal 45418 that was not infected with SIV, suggesting that there was no substantial SIV-induced change in the frequency of RA9-specific CD8 T cells (Figure 4c).

### Optimization of Mane A*10-RA9 Tetramer-ICS assay

Although the ICS assays allow us to study the functions of T cell responses to specific antigens, these assays are limited to only studying the cells that are producing effector molecules. As such they provide no information about antigen-specific cells that do not produce a response or may be dysfunctional. Incorporation of tetramer staining into the ICS assay allows us to home in on antigen-specific cells as a whole population and investigate how these cells are responding. However, a frequent problem that arises with the use of tetramers in ICS assays is the antigen induced down-regulation of the T-cell receptor (TCR) on antigen-specific CD8 T cells which greatly reduces the ability of tetramers to bind to these cells [47], [48].

**Figure 5:**
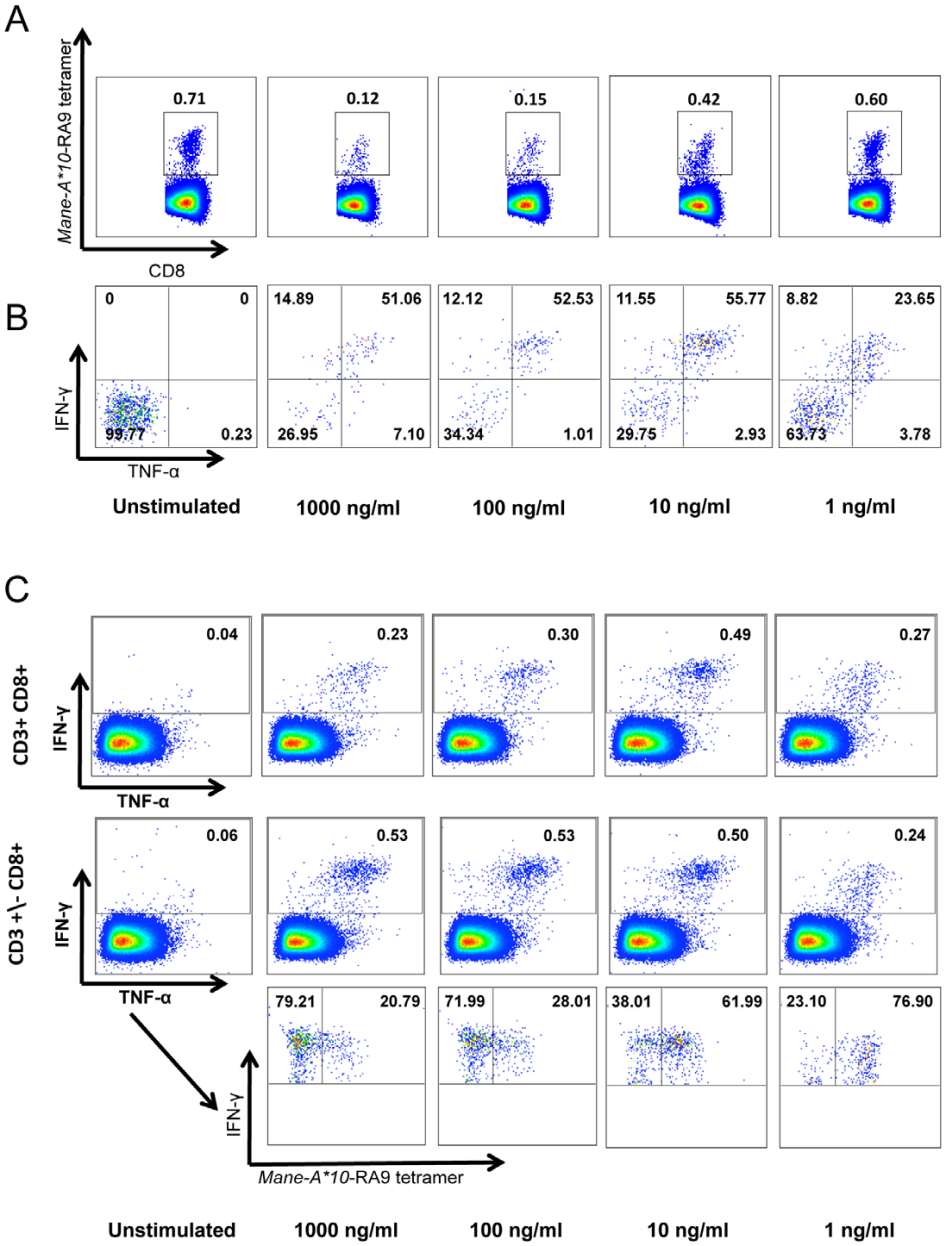
T-cell receptor down-regulation in response to antigenic stimulation. Tetramer-ICS assay performed using blood from an animal inoculated with influenza viruses (animal #26359) by stimulating blood cells with either DMSO (unstimulated) or decreasing concentrations of RA9 peptide (1000 ng/ml, 100 ng/ml, 10 ng/ml and 1 ng/ml). (**A**) Loss of tetramer+ cells following in vitro stimulation with high RA9 peptide concentrations. Cells were gated on CD3+CD8+ followed by gating on influenza RA9 tetramer positive cells. (**B**) Higher cytokine expression in influenza RA9-specific cells following stimulation with >10 ng/ml of RA9. The IFN-γ and TNF-α production from gated tetramer+ RA9-specific cells from panel (A) were compared between the decreasing concentrations of peptide. (**C**) Samples were analysed via two alternative gating strategies; gating on CD3+CD8+ cells, followed by gating on the total IFN-γ. Alternatively we gated on CD3 negative and positive cells (CD3+/−) and then gated on CD8+ cells, and measured the total IFN-γ production from these cells. Cells from this strategy were plotted as IFN-γ versus RA9-tetramer positive cells.

To optimize the use of the Mane-A*10-RA9 tetramer in an ICS assay we investigated the effect of stimulating the cells with 10-fold decreasing concentrations of RA9 peptide on the Mane-A*10-RA9 tetramer staining and RA9-specific CD8 T cell cytokine production (Figure 5a). A clear reduction in Mane-A*10-RA9 tetramer staining is observed as the concentration of the RA9 peptide antigen was increased; from a frequency of 0.71% in the unstimulated sample to 0.12% in the sample stimulated with 1000 ng/ml of RA9 peptide (Figure 5a). However, the frequency of Mane-A*10-RA9 tetramer positive cells producing IFN-γ and TNF-α remains similar (approximately 50%) for all RA9 peptide concentrations except 1 ng/ml where the frequency drops to 23.65% (Figure 5b).

Interestingly, we also observed distinct differences in the total IFN-γ production when gating on CD8 positive cells that were also CD3 positive compared to all CD8 positive cells regardless of their CD3 phenotype. At higher RA9 peptide concentrations (1000 ng/ml and 100 ng/ml) the frequency of IFN-γ producing RA9-specific CD8 T cells is greater when CD8 T cells are not gated according to CD3 phenotype (Figure 5c). Furthermore, when we looked at Mane-A*10-RA9 tetramer staining of these IFN-γ producing CD8 cells we observed that Mane-A*10-RA9 tetramer positive cells only represent approximately one quarter of the total IFN-γ producing cells at these peptide concentrations. Whereas at lower RA9 peptide concentrations (10 ng/ml and 1 ng/ml), the CD3 phenotype does not appear to affect the frequency of IFN-γ producing CD8 cells and the proportion of these cells that are Mane-A*10-RA9 tetramer positive cells is greater than 60%. Our results suggest that in conjugation with TCR down-regulation there is also an associated down-regulation of the CD3 molecules of antigen-specific cells in the ICS assay. The effects of TCR and CD3 downregulation were limited when the concentration of stimulating peptide was reduced to 10 ng/ml without impacting greatly on the effector molecule production. Further experiments on effector molecule expression on tetramer positive CD8 T cells were performed with 10 ng/ml antigen stimulation.

### CD8 T cell responses to influenza and SIV using tetramer-ICS assay after influenza infection

**Figure 6:**
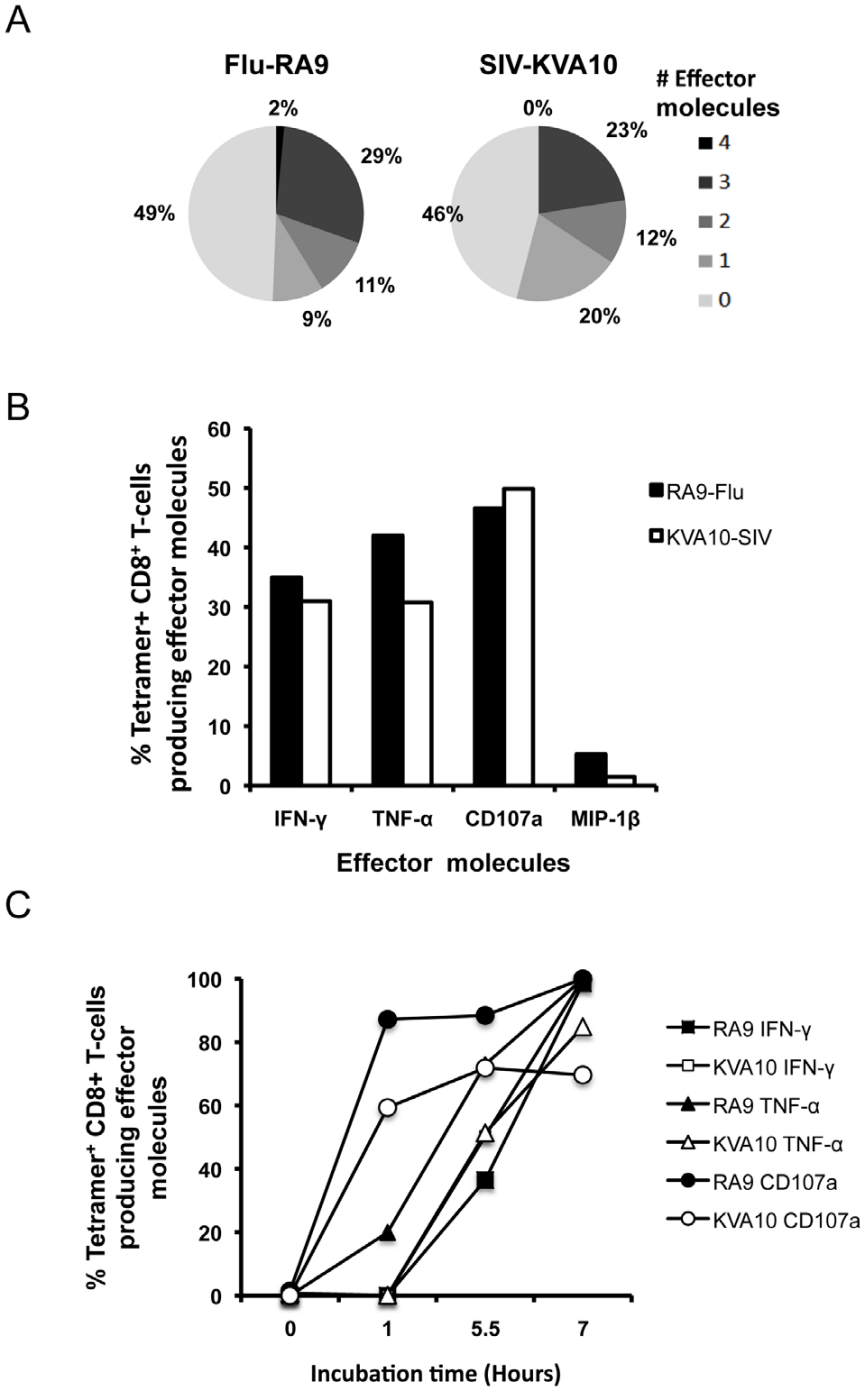
Comparison of tetramer positive influenza and SIV-specific CD8 T cell responses following recombinant influenza-SIV vaccination. Polyfunctional-tetramer ICS assay was performed on whole blood from day 0 post SIV infection stimulating with either RA9 or KVA10 peptides. (**A**) Summary of functional profile; the proportions of the number of effector molecules produced by antigen specific CD8 T cells when stimulated with either RA9 or KVA10 peptide. (**B**) The frequency of total effector molecules produced by RA9 (black) and KVA10 (white) specific CD8 T cells. Results are shown for 2 separate animals. (**C**) Comparison of the kinetics of effector molecule for RA9 (filled) versus KVA10 (open) at day 5 post SIV infection.

Having optimized the use of the Mane-A*10-RA9 tetramer in the ICS assay we used this assay to determine the polyfunctionality of influenza- and SIV-specific CD8 T cell responses after influenza-SIV vaccination. As seen with the standard ICS assay (Figure 2) the overall pattern of effector molecule expression from CD8 T cells in response to either influenza RA9 or SIV KVA10 were similar (Figure 6a and 6b). This is consistent with the epitopes being expressed from the same vector and consistent with other studies using vaccinia vectors [49], [50]. Interestingly, the incorporation of tetramer in the ICS assay also allowed us to determine that close to half of the RA9- and KVA10-specific CD8 T cells did not produce any of the studied effectors molecules (as shown by pale section of pie chart in Fig. 6a), with 3–15% producing one effector molecule and very few cells (<1%) producing all 4 effector molecules. These studies were repeated in another 3 animals with similar results, where the most common number of effector molecule expressed was none, 11–15% of cells expressed one and <1% producing all 4 effector molecules. The pattern of expression of effector molecule expression was also similar across another 3 animals, with MIP1β the least commonly expressed (<2% of total) and IFNγ, TNFα and CD107a all of reasonable expression levels (47.9%, 20.2% and 31.4% of total respectively).

The kinetics of the *in vitro* effector molecule response has been proposed to reflect the ability of CD8 T cells to rapidly respond *in vitro* [21]. We therefore analyzed the kinetics of the effector molecule response following recombinant influenza-SIV inoculation by stopping the assay after 1, 5.5 or 7 hours of antigen stimulation. Results are shown for one animal that had robust RA9- and KVA10 responses and allowed analyses or responses developing over time (Figure 6c). Again similar profiles were seen for both the RA9- and KVA10-specific CD8 T cell response, with CD107a being produced after just 1 hour of peptide stimulation, followed by IFN-γ and TNF-α detectable after 5.5 hours and further increasing after 7 hours of antigen stimulation. This kinetic study was repeated in another influenza-SIV vaccinated animal and a similar pattern of effector molecule expression observed in the influenza CTL responses, with CD107a expression clearly detected at 1 hr, but the TNFα/IFNγ response only detectable at 5.5 h and increasing through to 7 h after stimulation (data not shown).

### Polyfunctionality of influenza and SIV-specific CD8 T cell responses following SIV infection

**Figure 7:**
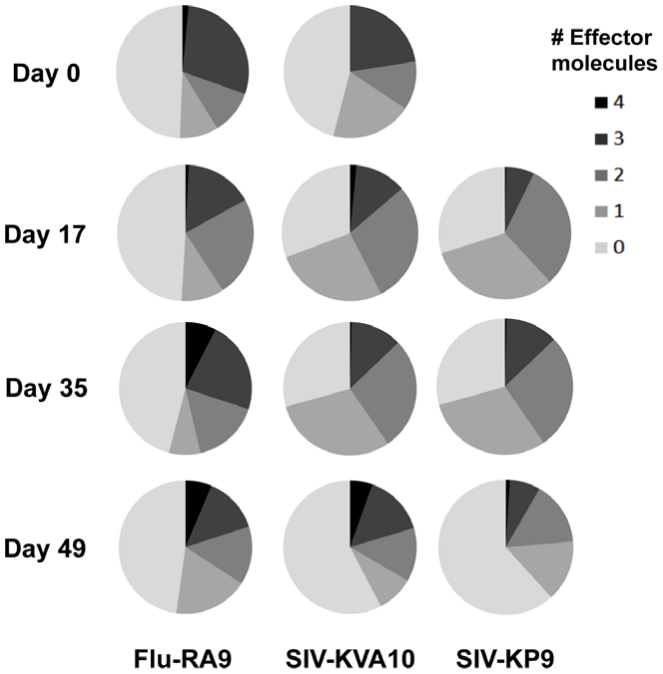
Comparison of polyfunctionality of influenza and SIV-specific CD8 T cells during SIV infection. Polyfunctional tetramer ICS assay was performed on whole blood from the animal 26359 at days 0, 17, 35, and 49 post SIV infection, stimulating with 10 ng/ml of either RA9, KVA10 or KP9. The pie-charts show the proportions of tetramer positive antigen-specific CD8 T cells producing 0–4 effector molecules. KP9-specific CD8 T cells were not detectable at day 0 post SIV infection.

The validation of the use of tetramers in the ICS assay allowed us to assess the polyfunctionality of the memory influenza-specific CD8 T cell response and the effector SIV-specific CD8 T cell response following acute and chronic SIV infection. The challenged animals all became infected with SIV_mac251_ after inoculation and had a mean peak SIV plasma RNA level at 2 weeks after challenge of 7.47 (±1.28) log_10_ copies/ml and a mean set point viral load from weeks 5–10 post-SIV infection of 6.29 (±0.25) log_10_ copies/ml. We analyzed in detail the proportion of CD8 T cells producing a number of effector molecules (IFN-γ, TNF-α, CD107a and MIP-1β) at different time-points following SIV challenge in fresh blood samples from one animal with robust influenza and SIV-specific CTL responses (Figure 7). Around the time of SIV challenge (and as discussed for figure 6a), the profiles of influenza RA9 and SIV KVA10 responses were similar (shown for comparison as the top pie charts in Figure 7). Throughout the first 7 weeks of SIV infection, the RA9-specific CD8 T cell polyfunctional response profile was relatively constant, with just under half of the RA9-specific CD8 T cells producing none of the studied effector molecules. In contrast, the SIV KVA10-specific CD8 T cell response became less functional by day 49 after SIV infection, with the proportion of KVA10-specific CD8 T cells not producing any effector molecules increasing to well over half of the tetramer+ cells through day 49 after infection. The reduction in effector molecule expression was largest for the CD107a+IFN-γ+TNF-α^+^ triple positive cells, which decreased from 28.5% of the tetramer+ population at day 2 to only 5.7% by day 49. Although no response was detected to SIV KP9 prior to SIV challenge, a similar trend was seen with the KP9-specific CD8 T cells during the course of SIV infection, which exhibited a less functional response at day 49 compared to the response at day 17. In another 3 animals studied for SIV KP9 CTL responses after challenge, the proportion of KP9-specific cells not expressing any effector molecules was also high at over 50% (range 55–75%) of the tetramer+ cells within 17–35 days after SIV infection reflecting poor effector molecule expression by SIV-specific CTLs after SIV infection.

### Sensitivity and avidity of influenza and SIV-specific CD8 T-cell responses following SIV infection

**Figure 8:**
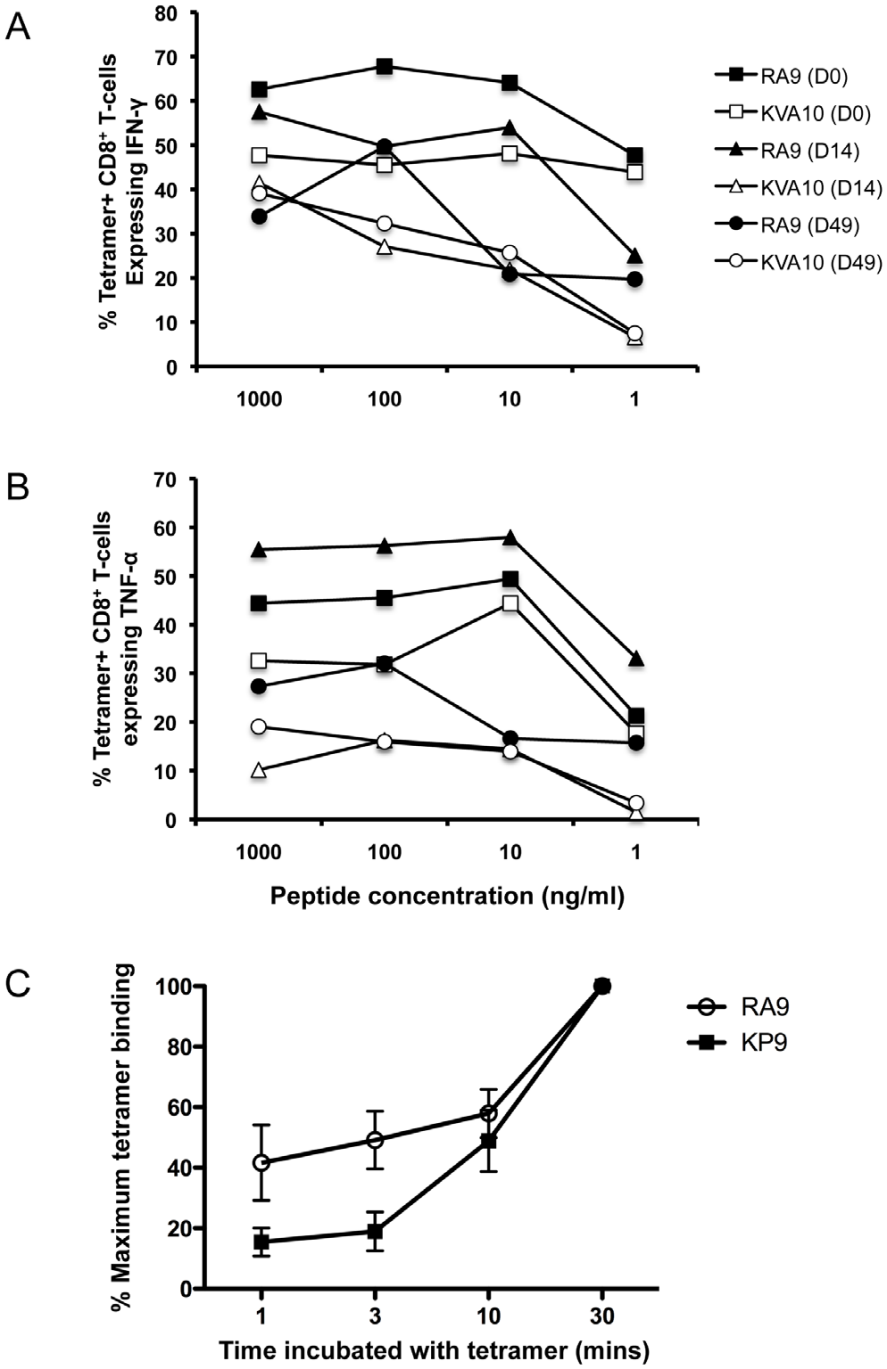
Comparison of sensitivity and avidity of influenza and SIV-specific CD8 T cells during SIV infection. (**A**) and (**B**) Comparison of re-stimulation induced cytokine production. An ICS assay was performed on whole blood from the animal 26359 at days 0, 14, and 49 post SIV infection, stimulating with decreasing concentrations of peptide (1000 ng/ml, 100 ng/ml, 10 ng/ml and 1 ng/ml) of either RA9 or KVA10 peptide. Function of the CD8 T-cells was assessed for IFN-γ expression (**A**) and TNF-α expression (**B**). (**C**) Comparison of relative levels of tetramer binding following incubation of PBMC samples from macaques with chronic SIV-infected that had been previously inoculated with influenza (n = 5) with either RA9-Mane-A*10 (circle) or KP9-Mane-A*10 (square) tetramer for 1, 3, 10 or 30 mins. Maximum tetramer binding was determined by the relative tetramer binding compared to 30 min incubated sample.

The ability of CD8 T cells to respond to low levels of peptide antigens has been shown to play a role in recovery from viral infections [13], [22]. This peptide sensitivity has been suggested to be an important aspect of CD8 T cell quality [51]. The quality of influenza- and SIV-specific CTL responses was therefore examined by assessing the CD8 T cell response after stimulation with different concentrations of peptide at different time-points following SIV infection in fresh blood samples from one animal with robust influenza and SIV-specific CTL responses (Figure 8a). Prior to infection (day 0), similar proportions of both influenza RA9- and SIV KVA10 tetramer+ CD8 T cells produced IFN-γ and TNF-α across a range of peptide concentrations (square symbols in figure 8b). The proportion of influenza RA9 tetramer+ CD8 T cells producing IFN-γ and TNF-α at low levels of antigen stimulation was maintained at high levels through to day 14 of SIV infection with a modest drop off by day 49 (closed symbols in figure 8b). In contrast, the SIV KVA10 tetramer+ CD8 T cells almost completely lose the ability to produce IFN-γ and TNF-α after low levels of antigen stimulation by day 14 and this is maintained through day 49 of infection. This experiment was repeated in an additional influenza-SIV vaccinated animal after SIV infection and we again observed a substantial reduction in cytokine expression by SIV KVA10 tetramer+ cells by day 14–49 after SIV infection expression at low peptide concentrations (data not shown).

Another important measure of virus specific CD8 T-cell response quality is the avidity of T-cells for their MHC/peptide tetramer prior to and following challenge. The avidity of influenza and SIV specific CD8 T-cells for their respective MHC-tetramer/peptide complexes was measured by the tetramer association assay that incubates PBMCs for different lengths of time in the presence of excess concentrations of MHC tetramer. Virus-specific CD8 T-cells that rapidly bind tetramer can be characterized as higher avidity than those that bind only after a longer incubation. We studied both influenza RA9 and SIV KP9 tetramer positive cells in the same PBMC samples from 4 influenza inoculated, SIV-infected macaques. In each case, the influenza-specific CD8 T-cells had significantly higher tetramer avidity compared to SIV-specific CD8 T-cells (Figure 8c. p = 0.028 at 3 minutes of incubation, Ranksum test).

### Exhaustion markers of influenza and SIV-specific CD8 T-cells following SIV infection

**Figure 9:**
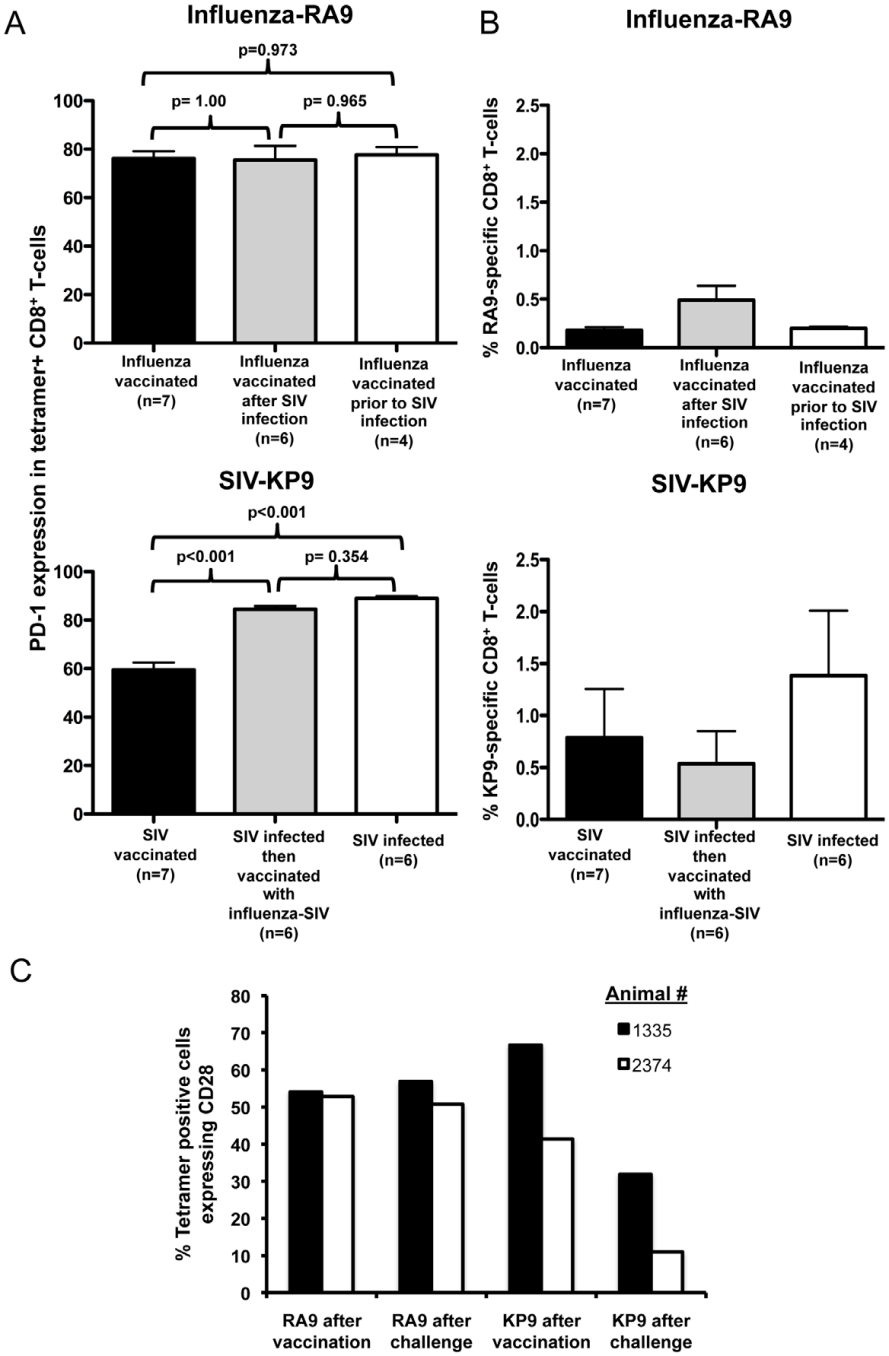
Exhaustion marker expression on influenza and SIV-specific CD8 T-cells. (**A**) Comparison of PD-1 expression on influenza RA9 tetramer-positive CD8 T-cells (top) and SIV KP9 tetramer-positive CD8 T-cells (bottom). RA9-tetramer staining was performed on frozen PBMC samples from animals either influenza vaccinated (influenza vaccinated after SIV infection or influenza vaccinated then SIV infected. Samples were typically tested at around 2–3 weeks post final vaccination. Tetramer staining of KP9-positive CD8 T-cells was performed on frozen PBMC samples from animals either, SIV vaccinated, SIV infected then vaccinated or SIV infected. Samples were compared using a One-way ANOVA with overall p = 0.956 for the influenza-RA9 comparison and p<0.001 for SIV-KP9 comparison (**B**) Magnitude of the tetramer responses in the same groups as for (B). Samples were compared as above using a One-way ANOVA overall p>0.05 for both influenza-RA9 comparison and SIV-KP9 comparison. (**C**) Frozen PBMC samples from two animals inoculated with recombinant influenza-SIV viruses (animals 1335 and 2374) were stained for CD28 expression on RA9 or KP9 tetramer positive cells prior to and following challenge with SIVmac251.

The difference in polyfunctionality and avidity of SIV-specific CD8 T-cells compared to influenza-specific CD8 T-cells could potentially be explained by the exhaustion of SIV-specific CD8 T-cells. The exhaustion of virus-specific CD8 T-cells has been shown previously to correlate closely with changes in expression of the marker PD-1 [24]. We therefore studied PD-1 expression on tetramer+ influenza- or SIV-specific CD8 T cells in PBMC from Mane-A*10+ pigtail macaques across a wide ranges of infection and vaccination scenarios. The PD-1 expression on influenza-specific CD8 T-cells from animals either influenza vaccinated, influenza vaccinated following SIV infection or influenza vaccinated then SIV challenged remained similar (Figure 9a). To study KP9 SIV-specific CD8 T-cells in the absence of SIV infection we analyzed stored PBMC from either recombinant influenza-SIV inoculated animals prior to SIV infection or from prior studies of DNA and fowlpox virus prime-boost immunization expressing SIV Gag prior to SIV infection [40], [45]. The PD-1 expression on vaccine-induced SIV KP9-specific CD8 T-cells was significantly less than on KP9-specific CD8 T cell from SIV infected animals (Figure 9a). Further, animals that received an influenza-SIV vaccination only after SIV infection also had a greater PD-1 expression than SIV vaccinated uninfected animals (Figure 9a). This suggests that the RA9 influenza-specific CD8 T-cell responses do not express the same amount of the exhaustion marker PD-1 compared to the KP9 SIV-specific CD8 T-cell response following SIV infection. For comparison, the magnitude of the influenza-specific and SIV-specific CTL responses at the times measured adjacent to the PD-1 expression was also assessed (Figure 9b). There were only modest non-significant differences in the magnitude of the responses across the groups.

The dysfunction and exhaustion of SIV-specific CD8 T-cells is likely explained by chronic stimulation of SIV-specific CD8 T-cells following SIV infection. One marker of chronic antigenic stimulation is loss of the expression of the costimulatory molecule CD28 [52], [53]. We therefore studied CD28 expression on influenza and SIV-specific CD8 T-cells in stored PBMC from animals inoculated with recombinant influenza-SIV viruses prior to and after SIV infection. We found that influenza-specific CD8 T-cells express similar levels of CD28 before and after infection with SIV (Figure 9b). Vaccine-induced SIV-specific CD8 T-cells express similar levels of CD28 to influenza-specific CD8 T-cells prior to SIV infection, but following infection CD28 expression on the effector SIV-specific CD8 T-cells decreases substantially (Figure 9b).

## Discussion

Improved primate models are needed to directly compare CD8 T cell immunity that assist in resolving acute viral pathogens and CD8^+^ T cell immunity that fails to resolve chronic viral pathogens within the one host. Macaques are a potentially useful non-human primate model to study important viral pathogens such as influenza and HIV. We recently developed an influenza-SIV vaccination model of Mane-A*10+ pigtail macaques [28] and used this to study both influenza-specific and SIV-specific CD8 T cells. A common influenza nucleoprotein-specific CD8 T cell response was mapped to a minimal epitope (termed RA9). The RA9 CD8 T cell response was restricted by Mane-A*10 and a Mane-A*10-RA9 tetramer was developed to study this response in more detail. We found that the memory RA9 influenza-specific CD8 T cell response following resolution of influenza infection, in comparison to effector SIV-specific CD8 T cell responses during chronic persistent SIV replication, maintained a highly functional profile in terms of the multitude of effector molecule expression, avidity and expression of exhaustion markers despite SIV infection.

The mapping of an influenza CD8 T cell epitope restricted by a common MHC I allele and generation of a MHC-I tetramer for this epitope is an important advance for influenza studies in macaques. The utility of a functional RA9 specific CD8 T-cell response to control influenza infection can now be analyzed in future studies of pathogenic macaque influenza infection. Interestingly, of the 4 CD8 T cell epitopes now known to be restricted by Mane-A*10 (RA9, KP9, KVA10, KSA10), only RA9 does not start with a double lysine (KK) motif [54]. We are planning structural studies to understand how all these epitopes are accommodated and presented by Mane-A*10. The development of a more refined motif for epitopes capable of binding to Mane-A*10 should enable scanning of viral genomes of influenza, SIV and other pathogens for other Mane-A*10-presented epitopes. An improved understanding of the presentation of Mane-A*10 epitopes should also lead towards more refined analyses of the evolution of viral escape mutations [33], [35].

Influenza-specific CD8 T cells have been extensively studied in mice and humans in recent years [55], [56] but non-human primate and ferret models of influenza have lagged behind in terms of sophisticated immunologic reagents [30], [57], [58]. The development of an MHC tetramer to track these cells in non-human primates is an important advance. We used the Mane-A*10-RA9 tetramer to track the influenza CD8 T cell response over time, enabling an analysis of cells that did not express any of the effector molecules studied. Around half of influenza-specific CD8 T cells we detected with the tetramer did not express any of 4 effector molecules after the vaccination process; these cells would not be detected by most standard Elispot and ICS techniques. We performed detailed kinetic and peptide-responsiveness assays on tetramer-positive cells within fresh macaque blood samples, although the conclusions for some analyses should be tempered by the technical difficulties in studying fresh samples from multiple animals concurrently. In general however, the functionality in terms of effector molecule expression of the SIV-specific CD8 T cells induced by the SIV epitope encoded within influenza was similar to that of the memory influenza specific CD8 T cells prior to SIV infection, but the chronically activated SIV-specific CD8 T cells rapidly became less functional within weeks after ongoing SIV infection.

Our results are in agreement with the multiple dysfunctions frequently observed in SIV-specific CD8 T cells in macaques and HIV-specific CD8 T cells in humans observed during the course of infection [12], [14]. The retention of functions by the influenza-specific CD8 T cells during early SIV infection was somewhat surprising given the broad immunodeficiency induced by SIV. Indeed it was recently shown that some influenza strains can be more pathogenic in HIV-infected humans compared to HIV-negative humans, suggesting influenza-specific immunity is impaired during HIV infection [59]. On the other hand, in our study the SIV-specific CD8 T cells are actively responding to a persistent viral infection whereas the influenza-specific CD8 T cells are no longer responding to an antigenic challenge. This could reflect dysfunction of the SIV-specific CD8 T-cells from chronic antigen-induced stimulation inducing the down-regulation of the T cell receptor observed after in vitro antigen stimulation. Future studies of influenza infection of SIV-infected macaques can now further analyze the effect of SIV infection on the developing influenza-specific CD8 T cell response. This has implications for the effectiveness of influenza vaccination in the setting of HIV infection [60].

The maintenance of a more functional profile by influenza-specific CD8 T cells despite SIV infection in this model likely reflects the efficient priming of this response and the lack of persistent antigenic stimulation [24], [52], [61]. Influenza-specific CD8 T cells had minimal or no increase in their expression of the exhaustion marker PD-1 in contrast to SIV-specific CD8 T-cells following SIV infection. In addition, expression of the CD28 costimulatory molecule expression decreased on SIV-specific CD8 T-cells but was maintained on influenza-specific CD8 T-cells. Taken together, the results suggest that chronic antigen stimulation has led to the dysfunctional SIV-specific CTL response but that in the absence of chronic antigenic stimulation the function of the memory influenza-specific CTL response is preserved.

In summary, this study develops important reagents to study influenza-specific CD8 T cells in pigtail macaques we show that effector SIV-specific, but not memory influenza-specific CD8 T cells, rapidly become dysfunctional during SIV infection. Our work suggests that vaccine or immunotherapy strategies to maintain more functional HIV-specific CD8 T cells during HIV infection should be of value in achieving better virologic control and delaying HIV disease.
